# Multicriteria optimization achieves superior normal tissue sparing in volumetric modulated arc therapy for gastric cancer

**DOI:** 10.1186/s12885-024-13067-y

**Published:** 2024-11-11

**Authors:** Ling He, Xinrui Gao, Tao Li, Xia Li, Xiaowen Sun, Zhigong Wei, Xingchen Peng, Jianghong Xiao

**Affiliations:** 1 Department of Biotherapy, Cancer Center, West China Hospital, Sichuan University, Chengdu, 610041 China; 2 Radiation Physics Technology Center, Cancer Center, West China Hospital, Sichuan University, Chengdu, 610041 China; 3 Department of Radiation Oncology, Cancer Center, West China Hospital, Sichuan University, No.37 Guo Xue Xiang, Chengdu, Sichuan 610041 China

**Keywords:** Gastric cancer, Multi-criteria optimization, Single-criteria optimization, Volumetric modulated arc therapy, Serial organ, Radiotherapy planning

## Abstract

**Objective:**

To evaluate the benefits of volumetric modulated arc therapy (VMAT) based on multicriteria optimization (MCO) for gastric cancer patients, particularly the protection of serial organs at risk (OARs) that overlap with the target volume.

**Methods:**

MCO and single-criterion optimization (SCO) VMAT plans were conducted among 30 gastric cancer patients, with a prescription dose of 50.4 Gy delivered in 28 fractions. All treatment plans underwent review, and a comparison was made between the active planning time and different dose-volume parameters.

**Results:**

Both the MCO and SCO VMAT plans achieved the target dose coverage, with no significant difference in the conformity index (CI) for the planning target volume (PTV), at median CI values of 0.887 and 0.891, respectively (*P* = 0.417). The MCO plans showed a slight but significant increase in the homogeneity index of the PTV, with a median increase of 0.029 (*P* < 0.001). Additionally, the MCO plans resulted in a lower *D*_*2%*_ to the small intestine and duodenum, with reductions of 3.43 Gy and 0.3 Gy, respectively (*P* < 0.05). Furthermore, the *D*_*max*_ to the small intestine correlated moderately with the overlapping volume between the small intestine and the target volume (ρ = 0.42, *P* = 0.023). Except for the mean dose to the liver, the MCO plans performed better in terms of dose indicators for other OARs. Moreover, compared to the SCO plans, the median active planning time in the MCO plans was significantly reduced by 53.2 min (*P* < 0.0001).

**Conclusions:**

MCO can effectively help the physicians to quickly select an optimal treatment plan for patients with gastric cancer. It has been shown that MCO VMAT plans can significantly reduce the dose to OARs and shorten the active planning time, with acceptable target coverage. In addition, these plans take less dosimetric time, thereby streamlining the treatment planning process.

## Introduction

Gastric cancer (GC) is a highly heterogeneous molecular and phenotypic disease, ranking as the third leading cause of cancer-related deaths worldwide [1]. In China, the age-standardized 5-year survival rate for GC is 27.4% [2, 3]. Current therapeutic strategies include curative surgery coupled with postoperative radiochemotherapy, which has been proven to decrease local recurrence rates and offer long-term survival benefits [4, 5]. Volumetric modulated arc therapy (VMAT) has been widely applied in radiotherapy for GC because of its capacity to enhance planning quality and shorten overall treatment duration [6, 7]. However, VMAT planning requires meticulous manual parameter tuning by dosimetrists and multiple optimization iterations, making the process highly dependent on the expertise of the dosimetrists and inherently time-consuming [8, 9]. Recently, multicriteria optimization (MCO) has emerged as a promising approach to address these challenges. MCO facilitates to generate a diverse set of plans with multiple solutions based on dosimetrist-defined optimization criteria, with each solution representing a Pareto-optimal plan. The collection of all Pareto-optimal plans constitutes the Pareto surface, allowing radiation oncologists to interactively navigate and achieve optimal dose distributions in real time [10]. Previous studies have indicated that compared with conventional single-criterion optimization (SCO), MCO plans can significantly reduce the active planning time and diminish the radiation dose to organs at risk (OARs) for various tumors, including oropharyngeal, lung, and prostate cancers [11–14].

The sparing of OARs, especially those that overlap with the target volume, is a significant challenge in radiotherapy planning. In MCO planning, Craft and colleagues have demonstrated that employing mean dose constraints is the most direct and effective approach for controlling the dose to OARs that overlap with the target volume [15]. For serial organs, however, focusing solely on the mean dose is inadequate, and it is also essential to limit the near-maximum dose, such as *D*_*2%*_ (where *D*_*V*_ represents the dose absorbed by the V% of the volume receiving the highest dose). At present, there is a lack of research on the strengths and weaknesses of MCO methods in these specific scenarios. Additionally, in GC radiotherapy planning, both the small intestine and duodenum may overlap with the planning target volume (PTV). To date, no studies have been reported to compare the dosimetric differences between MCO and SCO VMAT plans in GC patients. Furthermore, the potential of flattening filter-free (FFF) beams, which offer benefits such as high dose rates, reduced leakage, and lower out-of-field doses, has not been investigated in the scope of MCO VMAT planning for GC. Therefore, the objective of this study is to compare the differences in dose distribution to the target volume and OARs, and OARs sparing, particularly those overlapping with the target volume, between MCO and SCO approaches in GC FFF VMAT planning. The study also seeks to evaluate the potential advantages of MCO, especially in the protection of serial organs that overlap with the target volume. This investigation could provide valuable insights into optimizing radiotherapy plans for GC patients and enhance the application of MCO in the clinical setting.

## Materials and methods

### Patient selection

A total of 30 histologically confirmed GC patients who were treated with VMAT at West China Hospital, Sichuan University between May 2018 and December 2022 were enrolled in this retrospective study. All the patients had undergone curative resection (partial gastrectomy or near total/total gastrectomy) or with extensive (D2) lymph node dissection (if the pathological stages T3-4 or positive lymph node). The inclusion criteria were as follows: (1) receipt of postoperative adjuvant radiotherapy for GC; (2) a standardized radiation dose of 50.4 Gy delivered in 28 fractions; and (3) the use of VMAT as the radiation delivery technique. Patients were excluded if they had prviously undergone multiple courses of radiotherapy or if their radiation plan included multiple dose levels. The clinical characteristics of the patients are summarized in Table 1.

**Table 1:** Baseline characteristics of patients

Characteristic	No	%
**Age, year (median, range)**	54	30–75
**Gender**		
Male	21	70.0
Female	9	30.0
**ECOG performance status**		
0–1	12	40.0
2	18	60.0
**Site of primary lesion**		
Body of stomach	26	86.7
Cardia	4	13.3
**Pathological type**		
Adenocarcinoma	22	73.3
Signet-ring cell	8	26.7
**Stage (American Joint Committee on Cancer 7th revision)**		
IIB	6	20.0
IIIA	8	26.7
IIIB	9	30.0
IV	7	23.3
**Surgery type**		
Partial gastrectomy	13	43.3
Near total/total gastrectomy	17	56.7
**No. of lymph nodes dissected**		
Median (range)	24	13–81
**PTV volume (cc)**		
Median (Q_1~_Q_3_)	359.5	279.6–566.8

*Abbreviations*: *ECOG* Eastern Cooperative Oncology group, *PTV* Planning target volume, *Q1~Q3* stands for the first quartile to the third quartile

### Target structures and OARs

Patients were immobilized using a thermoplastic mask in the supine position with their hands raised above their head. Patients were required to fast for 3 to 4 h prior to the CT scan or radiotherapy. To prepare for the imaging, the patients drank 400–500 ml of water 30 min before the CT scan. Additionally, they drank 300 ml of a water solution containing a contrast agent to enhance the visibility of the gastric stump or small intestine during the CT simulation for body positioning. Enhanced CT scans were performed using the Revolution CT system (GE, Boston, USA) with the following settings: 120 kV, 90 mAs, and a slice thickness of 3 mm, covering a field of view of 512 × 512 mm. All the CT images were imported into the RayStation treatment planning system (RaySearch Laboratories AB, Stockholm, Sweden) for further analysis. A radiation oncologist delineated the gross tumor volume (GTV) and clinical target volume (CTV) within the planning system. The PTV was created by expanding the CTV with a 5 mm margin in all three dimensions. The GTV was defined by the clearly localized residual tumor lesions, whereas the CTV included the critical areas such as the anastomosis site, remnant, the tumor bed, and regions at high risk for lymph node involvement. Moreover, the oncologist contoured other OARs, including the spinal cord, small intestine (jejunum and ileum), duodenum, liver, and kidneys, following established contouring guidelines for upper abdominal organs [16].

### Treatment planning

All plans were generated in the RayStation treatment planning system (RaySearch Laboratories AB, Stockholm, Sweden) and delivered using the Elekta Versa HD linear accelerator (Elekta, Stockholm, Sweden), utilizing a 6 MV FFF photon beam and dual arc VMAT technique for optimal dose delivery. Two experienced dosimetrists, with comparable years of experience, were responsible for the radiation therapy planning. One dosimetrist, with 16 years of experience, designed the treatment plans following clinical conventions. Another dosimetrist dosimetrist with 13 years of experience designed the plans via the MCO approach.

**Fig. 1:**
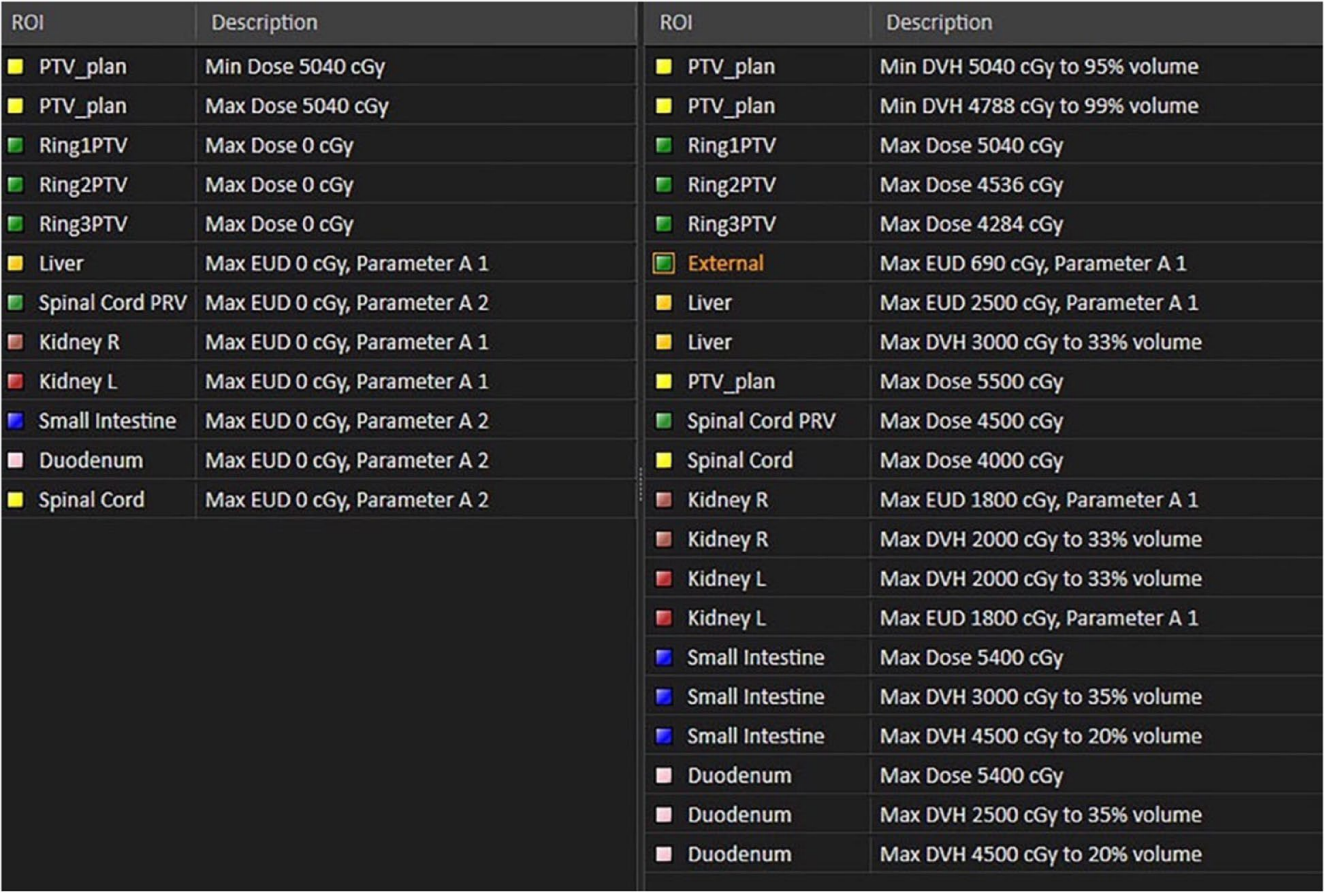
Objectives (left) and constraints (right) of MCO plans

The objectives and constraints for the MCO plans are shown in Fig. 1. The constraints for all OARs were set according to the NCCN Clinical Practice Guidelines for Gastric Cancer [17]. To minimize the dose to OARs, the constraints were appropriately adjusted due to the patient-specific anatomical relationship between target and OARs. For instance, before initiating Pareto optimization, the planning system identified violated constraints and computed the function value for each constraint. If the sum of the function values of all the constraints exceeded 1.00E-6, no feasible solution could be found. In such cases, constraints for the OARs with the highest objective function values were relaxed. If the MCO optimization indicated that a feasible solution could not be found, the constraints for the OARs with the highest objective function values were relaxed, followed by reoptimization until a feasible solution was obtained. On the basis of our previous study, a total of 60 Pareto plans were generated for each MCO plan in this research [11]. The Pareto optimization mode was based on fluence-based optimization process with a tolerance of 1.000E-5. The target priority for generating deliverable plans was set at 30, and the maximum number of iterations was set to 80. The number of pretranslation iterations was set at 7, and the leaf motion constraint was limited to 0.5 cm per degree. After the completion of MCO plan optimization, two experienced radiation oncologists navigated the Pareto surface. Each physician navigated 15 plans in a randomized double-blind mode, using SPSS software (version 23.0) to generate random numbers. Following navigation, further optimization was performed to obtain deliverable treatment plans. The navigation and optimization process could be repeated until satisfactory results were achieved.

### Plan analysis

The planning time for both plans was recorded, excluding the time spending on delineating targets and OARs. The Pareto plan generation time was recorded separately, as it does not involve active planner input. All the other human intervention planning time (active planner time) for both the SCO and MCO plans were recorded as “active planning time”. For SCO planning, all the time invested by the planner except review time by the physician was considered active planning time. For MCO planning, it included the time of setting up parameters, adjusting OARs and target structures, and postsegmentation adjustments, excluding physician review. The evaluation criteria for OARs adhered to the NCCN Clinical Practice Guidelines for Gastric Cancer [17]. The evaluation criteria for the target volume were set as follows: 95% of the target volume should receive at least the prescription dose of 50.4 Gy, *D*_*1%*_ should not exceed 110% of the prescribed dose, and *D*_*99%*_ should not be less than 95% of the prescribed dose. The conformity index (CI) and homogeneity index (HI) were used to assess the conformity and uniformity of the target dose. The calculation formulas for the HI and CI are based on the ICRU 83 report [18] and the Paddick index [19]. TV_PIV_ refers to the absolute volume of the PTV covered by the 100% prescribed dose. TV represents the absolute volume of the PTV, and PIV represents the absolute volume covered by the 100% prescribed dose within the patient's outer contour.

$$HI=({D}_{2\%}-{D}_{98\%})\div {D}_{50\%}$$

$$CI=(T{V}_{PIV}\times T{V}_{PIV})\div (TV\times PIV)$$

Statistical analyses were performed via SPSS Version 23.0 (Statistical Package for the Social Sciences, SPSS Inc., Chicago, IL, USA). Before the comparison, the Shapiro-Wilks test and Levene’s test were used to test the normality and homogeneity of variance of the data. Considering the nonnormal distribution of the OARs data, the Wilcoxon signed-rank test was employed to evaluate the differences in dose between the two groups of plans. The correlation between the data was measured through Spearman's correlation analysis. A significance level of *P* < 0.05 (two-tailed) was considered statistically significant. The dose parameters were presented by median values with interquartile ranges. To determine the differences between the SCO and MCO plan groups, the median of these differences (MD) was calculated by subtracting the SCO plan data from the MCO plan data.

## Results

The median active planning time for the MCO plans was 37.3 min (range 33.6–41.6 min), compared to 90.8 min for the SCO plans (range 86.0–97.9 min). This indicated that the manual planning time was significantly shorter for the MCO plans than that for the SCO plans, showing a reduction of 53.2 min for MCO plans (range 49.1–59.2 min, *P* < 0.0001). Both MCO and SCO plans achieved acceptable target coverage and met the clinical requirements for target volume and OARs in all patients. In terms of target coverage, the HI of the PTV in MCO plans increased by approximately 0.029, which was statistically significant (*P* < 0.001). However, there was no significant difference in the CI between MCO and SCO plans (*P* = 0.417), with median CI values of 0.887 and 0.891, respectively. The comparison of PTV doses between the two groups are shown in Table 2.

**Table 2:** Dosimetric comparison of PTV does for SCO and MCO plans

	**SCO** Median (Q_1_ ~ Q_3_)	**MCO** Median (Q_1_ ~ Q_3_)	**MD** Median (Q_1_ ~ Q_3_)	***Z***	***P***
**PTV**					
*D*_*1%*_ (Gy)	53.07 (52.59 ~ 53.35)	54.07 (53.87 ~ 54.48)	-1.19 (-1.42 ~ -0.64)	-4.508	< 0.001
*D*_*2%*_ (Gy)	52.94 (52.46 ~ 53.29)	53.85 (53.61 ~ 54.31)	-1.10 (-1.40 ~ 0.61)	-4.515	< 0.001
*D*_*50%*_ (Gy)	51.78 (51.54 ~ 52.00)	52.54 (52.21 ~ 52.77)	-0.75 (-1.10 ~ 0.45)	-4.206	< 0.001
*D*_*95%*_ (Gy)	50.40 (50.40 ~ 50.40)	50.40 (50.40 ~ 50.40)	0.00 (0.00 ~ 0.00)	inapplicabe	inapplicabe
*D*_*98%*_ (Gy)	49.72 (49.44 ~ 49.85)	49.12 (48.16 ~ 49.32)	0.53 (-0.80 ~ 0.36)	-4.782	< 0.001
*D*_*99%*_ (Gy)	48.98 (48.64 ~ 49.41)	48.06 (47.91 ~ 48.41)	0.89 (0.59 ~ 1.05)	-4.508	< 0.001
*HI*	0.064 (0.053 ~ 0.072)	0.093 (0.083 ~ 0.101)	-0.030 (-0.038 ~ 0.019)	-4.782	< 0.001
*CI*	0.891 (0.872 ~ 0.898)	0.887 (0.877 ~ 0.903)	-0.003 (-0.008 ~ 0.007)	-0.812	0.417

*Abbreviations*: *SCO* Single-criterion optimization, *MCO* Multi-criteria optimization, *PTV* Planning target volume, *D*_*V*_is the absorbed dose in V% of the volume, *HI* Homogeneity index, *CI* Conformity index, *MD* represents the median of the differences (MCO data minus SCO data), *Q1~Q3* stands for the first quartile to the third quartile

**Fig. 2:**
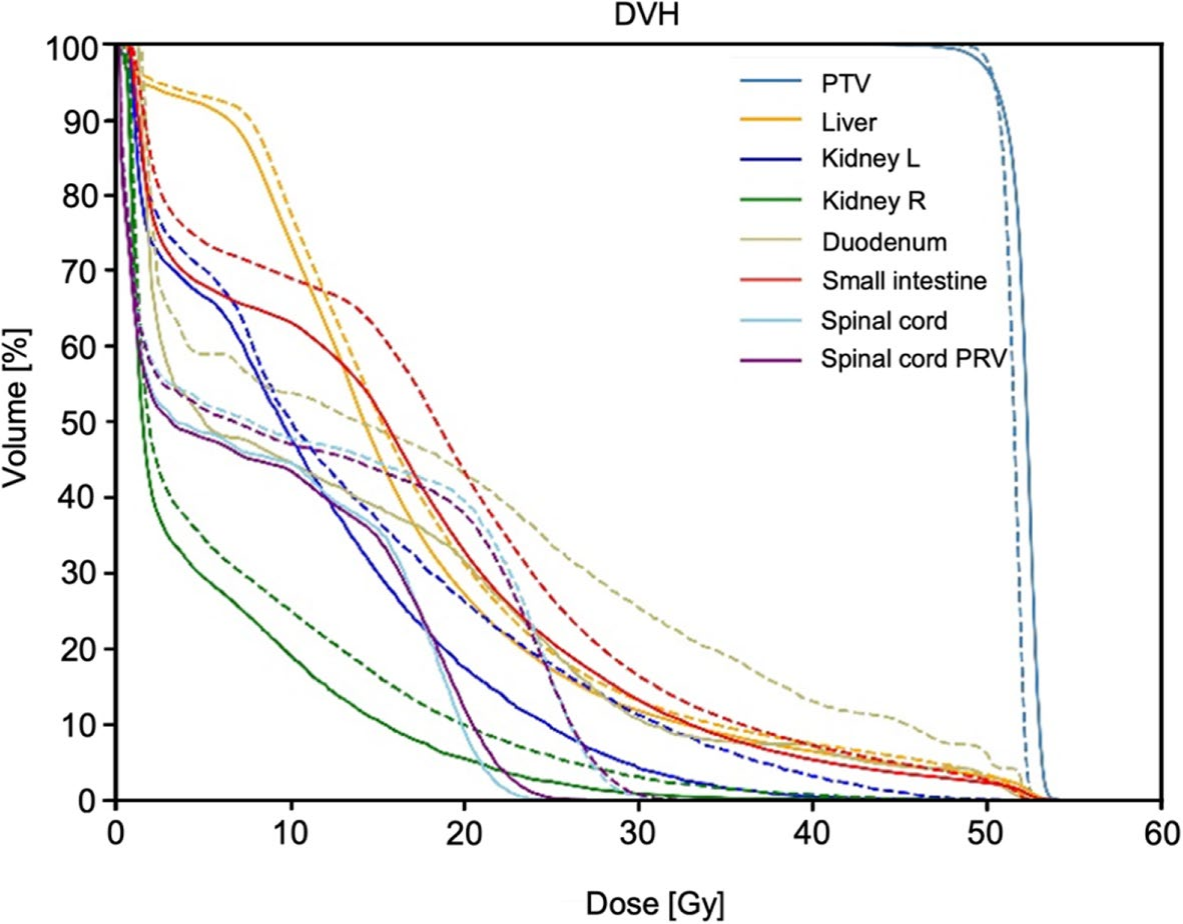
Comparison of dose-volume histograms between the SCO plan (dotted line) and the MCO plan (solid line) for a representative patient

**Fig. 3:**
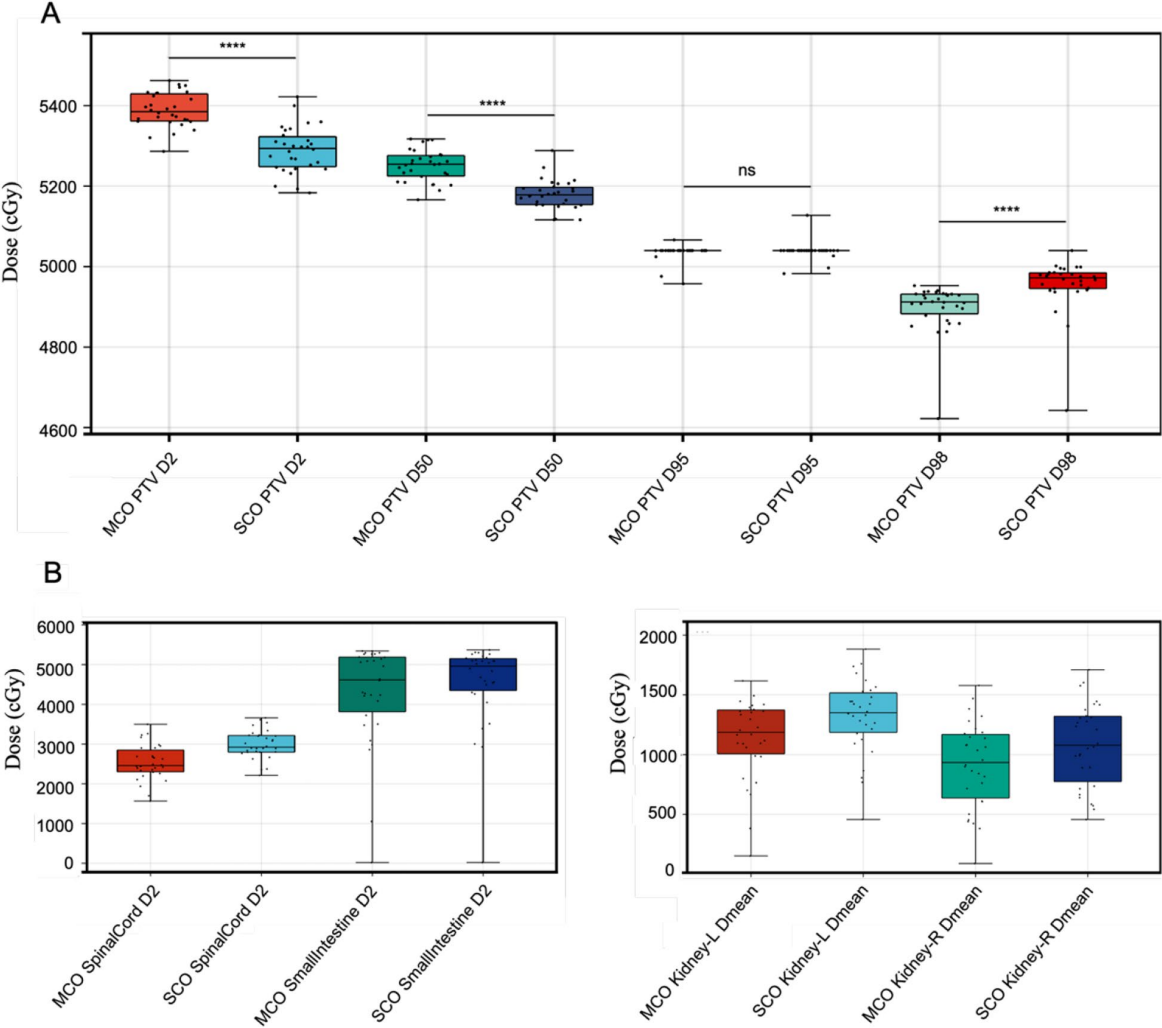
**a **Boxplots of D_2_, D_50_, D_95_, and D_98_ for planning target volume (PTV) dose distribution of SCO plans and MCO plans in 30 patients. Dv denotes the dose (Gy) of the PTV of v%. **b** Boxplot showing D_2_ and mean value of organs at risk (OARs) for SCO plans and MCO plans in 30 patients with gastric cancer

**Fig. 4:**
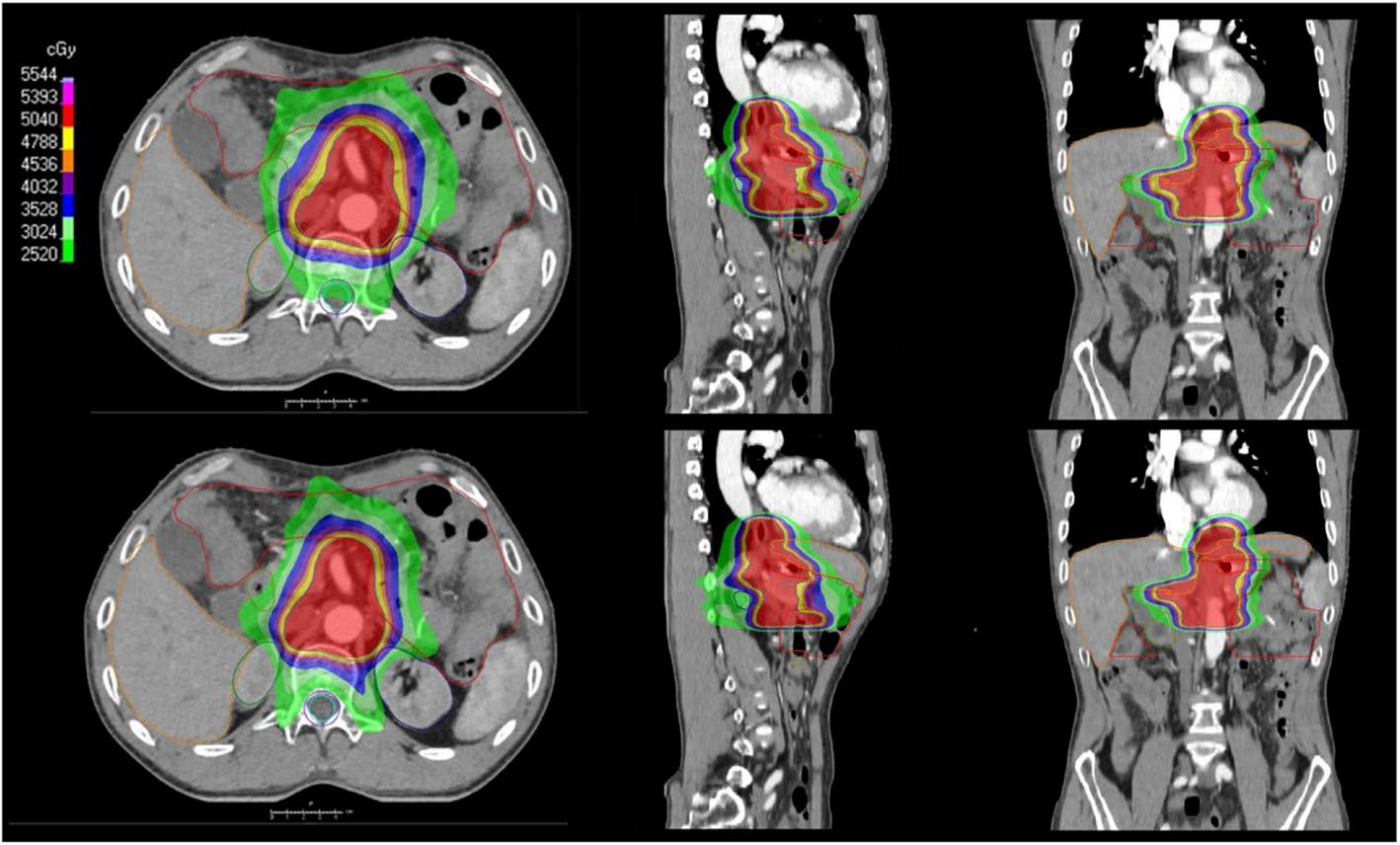
Transversal, sagittal, and coronal view of dose distributions for a SCO plan (up) and a MCO plan (down) of a representative patient

As shown in Table 3 and Fig. 2, regarding the OARs, the *D*_*max*_ and *D*_*2%*_ of spinal cord and spinal cord planning organ at risk volume (PRV) were significantly lower in MCO plans (*P* < 0.001). Compared with the SCO plans, *D*_*max*_ to the small intestine in MCO plans increased by approximately 1.17 Gy. In the MCO plans, the *D*_*2%*_ of the small intestine and duodenum decreased significantly by 3.43 Gy and 0.3 Gy, respectively (*P* < 0.05). Furthermore, MCO plans demonstrated a clear reduction in *D*_*mean*_, *V*_*15*_, and *V*_*20*_ for the kidney and *V*_*30*_ for the liver, compared to SCO plans (Fig. 3). This indicated an overall improvement in OARs sparing from higher doses of radiation. The *D*_*max*_ of small intestine revealed a moderate correlation with the overlapping volume between the small intestine and the target volume (ρ = 0.42, *P* = 0.023). Except for the liver *D*_*mean*_, the MCO plans generally resulted in lower doses for other OARs dose parameters than the SCO plans. Figure 4 displays the isodose line distribution and dose-volume histograms for a representative patient, comparing the two types of plans.

**Table 3:** Dosimetric comparison of the OARs for SCO and MCO plans

	**SCO** Median (Q_1_ ~ Q_3_)	**MCO** Median (Q_1_ ~ Q_3_)	**MD** Median (Q_1_ ~ Q_3_)	***Z***	***P***
**Spinal cord**					
*D*_*2%*_ (Gy)	29.22 (27.95 ~ 32.21)	24.56 (22.84 ~ 29.01)	4.54 (2.97 ~ 5.96)	-4.782	< 0.001
*D*_*max*_ (Gy)	31.68 (30.40 ~ 35.27)	27.61 (25.94 ~ 31.59)	3.67 (2.50 ~ 4.98)	-4.782	< 0.001
**Spinal cord PRV**					
*D*_*2%*_ (Gy)	30.60 (29.16 ~ 33.70)	27.58 (25.09 ~ 31.02)	3.60 (2.11 ~ 4.71)	-4.782	< 0.001
*D*_*max*_ (Gy)	34.58 (32.99 ~ 37.84)	32.63 (30.17 ~ 36.54)	2.39 (0.04–3.39)	-3.466	0.001
**Liver**					
*V*_*30 Gy*_ (%)	13.51 (10.98 ~ 16.50)	13.11 (11.16 ~ 16.20)	0.22 (0.14 ~ 1.11)	-1.080	0.280
*D*_*mean*_ (Gy)	16.21 (14.28 ~ 18.31)	16.39 (13.97 ~ 17.57)	0.46 (0.24 ~ 1.41)	-3.054	0.002
**Kidney L**					
*V*_*15 Gy*_ (%)	35.84 (27.91 ~ 40.06)	26.95 (19.46 ~ 32.97)	7.02 (3.41 ~ 11.12)	-4.714	< 0.001
*V*_*20 Gy*_ (%)	17.92 (14.15 ~ 22.59)	14.85 (10.54 ~ 18.64)	3.04 (1.39 ~ 6.12)	-4.618	< 0.001
*D*_*mean*_ (Gy)	13.51 (11.64 ~ 15.31)	11.87 (9.88 ~ 13.77)	1.32 (1.02 ~ 2.14)	-4.782	< 0.001
**Kidney R**					
*V*_*15 Gy*_ (%)	26.85 (16.12 ~ 34.31)	16.35 (10.85 ~ 23.96)	6.42 (3.04 ~ 9.73)	-4.782	< 0.001
*V*_*20 Gy*_ (%)	11.12 (8.33 ~ 16.60)	7.76 (4.56 ~ 11.26)	3.14 (1.12 ~ 5.67)	-4.782	< 0.001
*D*_*mean*_ (Gy)	10.78 (7.31 ~ 13.37)	9.35 (6.10 ~ 11.79)	1.32 (1.04 ~ 1.74)	-4.782	< 0.001
**Small intestine**					
*D*_*2%*_ (Gy)	49.60 (42.90 ~ 51.65)	46.17 (36.67 ~ 52.10)	0.54 (-0.22 ~ 3.79)	-3.054	0.002
*D*_*max*_ (Gy)	52.98 (52.67 ~ 53.80)	54.15 (52.99 ~ 54.45)	-0.78 (-1.47 ~ 0.02)	-2.062	0.009
*V*_*30 Gy*_ (%)	18.86 (9.28 ~ 27.79)	12.28 (5.37 ~ 23.47)	3.34 (1.31 ~ 5.68)	-4.660	< 0.001
*V*_*40 G*y_ (%)	6.86 (2.73 ~ 15.99)	4.34 (1.38 ~ 13.75)	1.28 (0.68 ~ 2.21)	-4.638	< 0.001
**Duodenum**					
*D*_*2%*_ (Gy)	50.92 (48.10 ~ 52.20)	50.62 (46.10 ~ 52.22)	0.37 (-0.20 ~ 2.25)	-2.499	0.012
*D*_*max*_ (Gy)	52.48 (51.97 ~ 53.19)	52.73 (52.03 ~ 53.66)	0.05 (-0.49 ~ 0.50)	-0.010	0.992
*V*_*25 Gy*_ (%)	39.84 (30.80 ~ 81.56)	31.12 (22.18 ~ 58.54)	6.63 (2.42 ~ 14.41)	-4.679	0.001
*V*_*35 Gy*_ (%)	24.88 (18.13 ~ 50.92)	18.73 (13.10 ~ 43.25)	4.69 (0.42 ~ 9.41)	-4.357	0.001

*Abbreviations*: *SCO* Single-criterion optimization, *MCO* Multi-criteria optimization, *OAR* Organ at risk,*PRV* Planning organ at risk volume, *D*_*V*_is the absorbed dose in V% of the volume, *D*_*ncc*_ the maximum dose to any point n cc or greater away from the PTV in any direction, *D*_*max*_ maximum dose, *D*_*mean*_ mean dose, *V*_*D*_ is the percentage of the OAR volume receiving ≥ D Gy, *MD* represents the median of the differences (MCO data minus SCO data), *Q1~Q3* stands for the first quartile to the third quartile

## Discussion

The quality of manually designed VMAT plans often depends on the dosimetrist's experience and is time-consuming [8, 9]. MCO can generate a variety of Pareto optimal plans, potentially leading to an ideal dose distribution for clinical radiotherapy [11]. Given the anatomical proximity of the small intestine and duodenum to the target volume in GC, they may overlap with the target volume, posing a challenge for radiotherapy planning, especially for serial organs. To our knowledge, no studies have reported the advantages and disadvantages of the MCO VMAT plan in terms of the near-maximum dose limitation for serial organs. Thus, this retrospective planning study was conducted to investigate the dosimetric benefits of MCO plans for GC patients and the feasibility of protecting serial organs that overlap with the target volume. We found that MCO plans not only significantly reduce the dose to OARs but also shorten the manual planning time while satisfying the target coverage. Interestingly, we also discovered that for serial organs overlapping with the target volume, although MCO does not show advantages in improving *D*_*max*_, it does significantly reduce *D*_*2%*_.

The critical determinant of local tumor control is the minimum dose delivered to the target volume [20]. In this study, based on the constraints of the MCO plans, we strictly hold the precision, ensuring that the *D*_*99%*_ was not less than 95% of the prescribed dose, and the *D*_*95%*_ was normalized to 100% of the prescribed dose. The results showed that for all patients, the MCO plans met the requirements for *D*_*99%*_ and *D*_*95%*_, and *D*_*1%*_ was maintained at less than 110% of the prescribed dose. Additionally, although the MCO plans exhibited a slightly poorer HI compared to the SCO plans, this finding is consistent with our previous study on oropharyngeal cancer and MCO [11]. Nevertheless, considering the *D*_*99%*_, *D*_*95%*_, and *D*_*1%*_ results, we believe that the MCO plans can meet the requirements for target coverage in GC.

The anatomical complexities of GC often result in the PTV overlapping with adjacent organs such as the small intestine and duodenum. Therefore, it is challenging to restrict the near-maximum dose to both the target volume and the overlapping organs. According to our previous study, the constraint settings may be pivotal in influencing the optimization results of MCO plans [11]. Craft et al. suggested that using the mean dose as a constraint for OARs that overlap with the PTV may be the most direct and effective approach in MCO plan design, rather than relying solely on the maximum dose [15]. However, for serial organs, limiting the maximum dose is obviously more important. In light of this, our study still used the maximum dose as a constraint for the small intestine and duodenum in the MCO plans. Compared with the SCO plans, the MCO plans either maintained or showed an increase in the median *D*_*max*_ for the small intestine and duodenum. Spearman correlation analysis revealed a significant correlation between the overlapping volume of the small intestine and its *D*_*max*_, which is in line with the findings of Craft et al. Conversely, the median *D*_*2%*_ slightly decreased for both the small intestine and duodenum. On the basis of these findings, we believe that for serial organs overlapping with the target volume, using the maximum dose as a constraint in MCO planning may not significantly improve the *D*_*max*_, but it does effectively improve the *D*_*2%*_. That extends the findings of Craft et al. Previous research indicated a direct relationship between radiation-induced intestinal injury and the *D*_*max*_ to the intestine, with higher doses increasing the risk of such injury [21]. Considering that ICRU Report 83 recommends using the near-maximum dose instead of the maximum dose [22], our strategy of reducing the *D*_*2%*_ dose may also serve to lower the probability of radiation-induced injury. Furthermore, studies have identified *V*_*40*_ and *V*_*25*_ as predictors of intestinal toxicity in the small intestine and duodenum, respectively [23, 24]. In our study, compared to the SCO plans, the median *V*_*40*_ for the small intestine and the median *V*_*25*_ for the duodenum were reduced by 1.2% and 6.6%, respectively. This reduction may contribute to a lower incidence of intestinal toxicity. In summary, for serial organs that overlap with the target volume, MCO plans, despite not showing superiority in improving *D*_*max*_, can effectively lower *D*_*2%*_ and other dose parameters. Consequently, it has the potential to decrease the risk of complications in the small intestine and duodenum in GC radiotherapy.

Radiotherapy for GC patients may result in complications of liver, kidney, and spinal cord injuries, which are directly related to the radiation dose [25–27]. Studies have shown that cumulative dose is a critical factor in the incidence of radiation-induced kidney injury. For instance, a cumulative dose of 18 Gy to 23 Gy correlates with a 5% incidence rate over 5 years, which turns to 50% when the cumulative dose reaches 28 Gy [27]. In our study, we observed that compared with the SCO plans, MCO plans realized a significant reduction in the *D*_*mean*_ for both kidneys. Specifically, the *D*_*mean*_ of the left kidney decreased from 13.51 Gy to 11.81 Gy, and that of the right kidney dropped from 10.87 Gy to 9.35 Gy. In addition, the median *V*_*15*_ and *V*_*20*_ of both kidneys were reduced by 3.0% to 7.0%. These findings demonstrate that MCO plans may probably lower the risk of radiation-induced kidney injury. Regarding the spinal cord, the median *D*_*2%*_ and *D*_*max*_ were reduced by 4.54 Gy and 4.34 Gy, respectively. While the threshold for a significant increase in the probability of radiation-induced spinal cord injury is 45 Gy [25, 26, 28], and all doses in this study were below this threshold for both MCO and SCO groups, the reduction of more than 4 Gy in doses exceeding this level may still contribute to spinal cord protection. In terms of the liver, although the *D*_*mean*_ of the liver in the MCO plan showed a slight reduction, we hold that it still has a positive effect on liver protection. In a comparative study by Hu et al., they examined the dosimetric differences between MCO plans in IMRT and SCO plans in VMAT for GC. They reported that MCO effectively reduced the *D*_*mean*_ and *V*_*15*_ of the kidneys [29]. However, their study revealed no significant differences in *D*_*max*_ of the spinal cord, *V*_*20*_ of either kidney, or *D*_*mean*_ of the liver between the two plans, contrasting with our findings. The main reasons for these discrepancies could be that their MCO plan utilized 6-field IMRT, whereas our MCO plan utilized full-arc VMAT. Full-arc VMAT has an advantage over IMRT of modulating the dose to OARs from all angles, which is limited by a smaller number of beam angles. Given the larger volume and distribution range of the liver and kidneys, IMRT beams are more likely to pass through the organs, resulting in an increased dose and less noticeable differences between the two plans. Additionally, their study compared MCO IMRT with SCO VMAT, introducing extra differences beyond the use of MCO. As the radiation therapy techniques were also different, both factors could influence the dose distribution. In contrast, our study compared MCO VMAT with SCO VMAT, which better illustrates the impact of MCO on dose distribution. Therefore, we propose that the use of MCO in VMAT can improve the dose distribution to the liver, kidneys, and spinal cord in GC patients. This optimization can help to reduce the probability of treatment-related complications for patients undergoing radiotherapy that involves these critical organs.

Planning efficiency is also one of our key concerns. In this study, the median planning time of the MCO plan was reduced by 53.2 min compared with that of the SCO plan. This finding aligns with those of studies conducted by Craft [15], Kamran [12], and Kierkels [30], who reported reductions in the mean planning time from 88 to 162 min with MCO. Although the time reduction was relatively modest in our study, it can be attributed to the differences in tumor location, which affects the complexity of each plan. Besides, the calculation speed of different planning systems may also a factor. Nevertheless, the median mean active planning time of the MCO plans in our study remained lower than that of the SCO plans, demonstrating that MCO can not only improve planning efficiency but also ensure the quality of the plans.

There are several limitations in our study. First of all, it is a single-center retrospective study, which inherently comes with certain constraints. Secondly, the MCO plans were not carried out in clinical practice, limiting our ability to assess their actual clinical efficacy and potential side effects. To address these limitations, more larger cohort and further prospective multicenter clinical studies are warranted to validate the dosimetric differences and treatment outcomes between the MCO and SCO plans specifically for GC patients. Such studies will be instrumental in validating the potential benefits of MCO in clinical practice.

## Conclusions

Taken together, MCO in VMAT planning for GC has shown promising results. Our findings indicate that MCO can effectively reduce the dose to OARs, including the small bowel and duodenum, with maintaining adequate target coverage. While MCO does not display an advantage in improving maximum doses to serial organs, it does show feasibility in reducing near-maximum doses. However, these findings are based on our specific study context and may require further validation. We believe that future prospective multicenter clinical studies will confirm the impact of MCO on patient outcomes. This will be crucial in investigating the benefits of MCO VMAT plans and exploring the extent to which they can improve the treatment of GC patients.

## Data Availability

The data used and analyzed in this study are available from the corresponding author upon reasonable request.

## Abbreviations

GC: Gastric cancer
VMAT: Volumetric modulated arc therapy
MCO: Multi-criteria optimization
SCO: Single-criterion optimization
OAR: Organ at risk
PTV: Planning target volume
CTV: Clinical target volume
FFF: Flattening filter-free
CI: Conformity index
HI: Homogeneity index
PRV: Planning organ at risk volume
MD: Median of the differences
